# Pandemic anxiety and its correlates among young doctors working frontline in Pakistan

**DOI:** 10.1017/gmh.2020.22

**Published:** 2020-09-30

**Authors:** Alina Rashid, Kanza Faisal

**Affiliations:** 1 Shaukat Khanum Memorial Cancer Hospital and Research Centre, Lahore, Pakistan; 2 Institute of Applied Psychology, University of the Punjab, Quaid-e-Azam Campus, Lahore, Pakistan

**Keywords:** Challenges and Threats, COVID-19, Frontline Workers, Pakistan, Pandemic Anxiety

## Abstract

**Background:**

The study explores the challenges and threats young doctors in Pakistan working frontline toward the pandemic face, and how it translates into their personal and social lives.

**Methods:**

Thirteen resident doctors working frontline with COVID-19 cases were approached and interviewed in-depth until the point of data saturation. Interpretative phenomenological analysis was used as a method for analysis.

**Results:**

Four themes emerged in the analysis: pandemic anxiety, organizational lack of readiness for change, impact on interpersonal relationships, and commitment to service. Young doctors experienced psychological distress, and emotional vulnerability due to the challenges and concerns faced by them in the wake of COVID-19. Shortage and excessive reuse of personal protective equipment, direct exposure to the disease, concern for personal safety, fear and apprehension of being a probable disease carrier for families, physical distancing from loved ones, long working hours, and increased organizational responsibility altered the quality of life for doctors working frontline toward pandemic in COVID wards. Doctors who received appreciation and support by families reported improved filial bonds.

**Conclusion:**

The adverse effect on psychological health, personal and social life, and increased professional demands have been some of the major challenges and threats faced by young doctors working frontline toward the pandemic. However, unconditional love and support from family and community has proven to reduce pandemic anxiety among doctors. Furthermore, the sense of compassion and the satisfaction in service to community has kept doctors devoted to fight against pandemic 2020.

## Introduction

World Health Organization declared COVID-19 or the new coronavirus as a pandemic in March 2020. The struggle for survival over resources in the wake of COVID-19 became a cosmopolitan issue. Pakistan being an underdeveloped country, struggles to contain and reduce the burden of this illness before it outgrows the resources available to fight the deadly virus (The Nation, 2020). The government is helping its people to understand the need to take precautionary measures, now, more than ever (Government of Pakistan, 2020). With hospitals strained and the number of fatalities rising dramatically on a day to day basis, Pakistan's fragile health sector is bracing for an impending epidemic. Hospitals are still failing in the two most populated cities of Pakistan, Karachi and Lahore, due to a significant rise in COVID-19 patients in recent weeks. Many government and private hospitals had to turn away patients due to the shortage of beds and ventilators. In Karachi, home to more than 15 million people, the government, private and not for-profit hospitals are grappling with coronavirus patients. The health minister of Pakistan, Yasmeen Rashid, has declared that there are only 539 beds and 200 ventilators for patients with coronavirus in Punjab (Government of Pakistan, 2020).

Having only 2000 ventilators country wide, the stress among general population and the need for medical institutes to cater for the patients suffering with this deadly disease is expected to be insufficient (The Nation, 2020). With the increased need of treatment provision, the burden comes on the shoulders of frontline paramedic staff. Not only do they bear the burden of treatment provision but have also been reported experiencing anxiety and hopelessness (Shaw, 2020), and in grim need of psychological support in many countries (Banerjee, 2020). Restricting the exposure to disease and reducing the additional stress of unprecedented circumstances such as COVID-19 on the shoulders of experienced and seasoned practitioners, young doctors are facing the challenge to directly manage patients in COVID wards. Therefore, this study explores what it means for young doctors in Pakistan to work frontline toward pandemic, to get in-depth understanding into their psychological, personal, social and occupational state. Such insights will help in understanding the functioning of doctors during unique crisis like a pandemic.

## Method

### Research design

Interpretative phenomenological analysis (IPA) focuses on the lived experiences of participants. It considers both phenomenology (i.e. the participant's attributed meaning of events, experiences, and states) and interpretation (i.e. the researcher's conception, belief, expectation, understanding, and reporting of participant's experience) (Smith *et al*., 1999). An idiographic case study approach was used to make sense of the data. Themes shared between cases were explored. The analysis reflected upon the subjective views of participant's experiences and the reflexive reporting of those experiences by the researcher (Willig, 2001). Inductive approach in IPA allowed the unanticipated to emerge and provided an in-depth and holistic perspective (Smith and Osborn, 2003; Smith, 2004).

### Participants

Through snowball sampling strategy, data were obtained until the point of saturation from 13 participants (nine male and four female doctors). Participants were resident doctors aged between 25 and 32 years, working frontline in managing and treating COVID-19 patients in the designated COVID wards. Out of these 13 participants, seven resided in Lahore, three in Karachi, two in Gujranwala, and one in Sargodha.

### Procedure

Due to the sensitivity of situation and the need for physical distancing, interviews were conducted online through Zoom after coordinating with the participant's availability, to reduce the chance of dropouts. Informed consent was taken verbally notifying participants about the interviews being audio recorded and their right to voluntarily participate and leave the study at any point for any reason. Confidentiality and anonymity were assured. Semi-structured interviews based on an interview schedule that broadly addressed the personal, professional, social, and psychological concerns and coping were open-endedly asked. All participants were interviewed by the first author. Participants could respond bilingually (in English and Urdu) allowing for open and free expression of their experiences. However, all transcriptions were translated into English language. Two-rater coding was independently done by both authors to increase the reliability of the study. To ensure credibility, respondent validation was done by sending back the analyzed data to the respondents for accuracy. Participants agreed that the results resonated with their experiences and hence no changes were made. Data were anonymized at this point. The mean interview duration was 25 min.

## Results

Based on IPA, four themes were found.

### Pandemic anxiety

Stress, fear, worry, apprehension, uncertainty, changes in sleeping and eating patterns, agitation, difficulty in concentration, and work-related anxiety due to COVID-19 can be termed as pandemic anxiety. Fear of catching the disease and dying has induced great psychological distress. Social distancing, quarantine, and lockdowns are having an adverse effect on mental health. With responsibility of managing COVID wards being put on resident doctors, the exposure to patients tested positive for corona virus has instilled anxiety among the resident doctors as one participant said:

It causes a constant state of worry and panic at the back of my mind.

Doctors go through rigorous training and experience in dealing with patients suffering from different diseases. The risk for them to contract any disease is always present. However, COVID-19 is different, it 's a pandemic with quick transmissibility and no known treatment or cure yet. Young doctors reported facing extreme emotional vulnerability working frontline with COVID-19 patients, as it incites cognitive dissonance of putting aside the concern for personal safety to fulfill professional obligation. Having the state of confusion and to decide if feeling anxious during pandemic is alright or not, induced fear, apprehension, and agitation has been evident from the following:

I feel that I'm more emotionally vulnerable and anxious.

Moreover, all young doctors felt overwhelmingly concerned and pressurized about exposing their loved ones to the virus. This idea affected the quality of their mental and socio-occupational life. Huang and Zhao (2020) reported young health care workers were spending too much time fighting the pandemic that they are now at a higher risk for developing a mental illness. One of the participants while referring to how he has been experiencing life during pandemic responded as follows:

I can't go out; I'm not able to spend time with my friends. My social life is restricted. It (pandemic) has taken a toll on my mental health. I'm trying to cope with it, but this really affects me.

### Organizational lack of readiness for change

Organizational readiness is a shared psychological state in which members of an organization value and favor the implemented task demands, resource availability, and situational factors (Weiner, 2020). In the wake of a pandemic, all organizations have changed their working, severely lacking effectiveness in terms of staff, equipment, funds, structure, and availability of resources. Most hospitals have lacked funds and equipment for meeting the challenges of pandemic. In some cases, the doctors were provided with equipment that was not properly sealed or due to the shortage of supply were asked to use the same personal protective equipment (PPE) multiple times. One of the participants highlighting the concerns, said:

I think the problem of adequate equipment, the organizational structure, infrastructure, and the lack of funds and provision of resources is the biggest problem that we have. What I struggle most with, is the lack of personal protective equipment and the limited availability of specialized doctors.

The lack of resources and capacity to cater to the pandemic consequences have put a lot of pressure on young doctors having to work long shifts, distorting work–life balance. However, to cater for the effects of coronavirus, some organizations have brought forth resident doctors from all specialties to work frontline with COVID-19 patients, impeding their professional field specific training. Participants felt the need for supervision by a senior doctor do discuss about COVID-19 patients was lacking as highlighted by one of the participants:

There's no senior doctor available. If you, being, an eye surgeon, is working with COVID patients then you at least need a senior medical consultant to supervise you all the time, which we don't get. Basically, SRs are working in emergency. You can only call them. If there is an emergency, and it takes them 20 minutes to get from emergency ward to COVID ward since the distance between the two wards is that much. No senior is immediately available for case discussions, if needed. The organization's working is very poor.

Moreover, organizations lacked incentives for essential frontline workers risking their lives for others. All these aberrant organizational changes made working with COVID-19 patients, an onerous task for resident doctors.

### Impact on interpersonal relationships

Despite the growing stress, fear and anxiety, the effects of pandemic on interpersonal relationships have been mixed for resident doctors. While this holds true for young doctors that with the increased work pressure and social distancing, their social life and social relationships were noted becoming unsettling and disturbed. However, family bonds were noted getting strengthened, participants reported receiving more appreciation, support and care from their families during this crisis. Regard for kinship increased. Communication and expression of love became more evident within the family structure improving interpersonal relationship with family as mentioned by one of the participants:

My father and my niece went up the house and they displayed that poster in support and solidarity with me and other doctors, it was very touching moment. I think that my family and friends are really behind me and that helps me.

However, in some cases, the families being overly concerned about the safety of the participant pressurized them to quit their job. Reports on the effect of COVID-19 on relationships indicate an increased number of quarrels (Bair and Czink, 2020) as supported by one of the participants while mentioning

Having to fight with your loved ones on daily basis to let you go for your duty because they're concerned for your safety.

Overall, resident doctors working frontline during pandemic felt that COVID-19 impacted their interpersonal relationships. However, most doctors reported receiving emotional support from their families.

### Commitment to service

Despite all the safety concerns and disruptive quality of life, young doctors devoted themselves to service. Resident doctors reported fighting against the pandemic as paying forth to their community. Doctors believed that even though the fight against pandemic is more than their professional duty, saving lives, and playing a substantive role during these challenging times has brought great sense of gratification and benevolence to them as evident from the following:

I'm a doctor and I have a certain skill and I have the obligation to perform, Call of Duty.

For me, I want to make a difference and reach out to those who need help in time of crisis. Standing up as a person, as a professional, as a health care worker, frontline healthcare worker, so that is something like a personal goal and professional demand. It brings a lot of gratification.

Getting appreciation and support from loved ones and people in general, appeared to make fighting against COVID-19 meaningful and satiating as evident from the following verbatim of the participant:

We get huge respect from community, from the security personnel, police, even generally. If you tell anyone that you are a doctor going on duty treating the COVID patients then they all are veneration towards you and give you lots of prayers.

Similarly, another participant reported:

I think I'm fortunate that most of the people that are here, almost all around me, have been very supportive. They understand my needs and have extended support to me, shared their space with me for me to feel comfortable, and, I haven't experienced any avoidance from them.

Acknowledgement of their services, appreciation and support of family, friends and public in general served as a primary incentive for doctors, reducing anxiety and motivating them in their fight against COVID-19.

## Discussion

This study explored how working frontline in the wake of COVID-19 translated for young resident doctors and what effect it had on their personal, professional, psychological, and social life. From getting concerned for personal safety due to the lack of PPEs to organizational challenges of not having enough supervision to cater to the needs of patient tested positive for coronavirus, resident doctors faced a large amount difficulties instilling in them fear, anxiety, apprehension, as well as agitation leading to disruptive sleeping and eating patterns. Similar trends in mental health have been reported where everyday strain due to extended job shifts, inadequate PPE and fear of infection as well as infecting their families were reported taking a toll on psychological, as well as physical health of frontline workers (Lancet, 2020).

These finding have been supported by Goyal *et al*. (2020), explaining how the fear of carrying and spreading the illness to other individuals made the nurses among other paramedic staff commit suicide within a few days of being exposed to suspected patients.

In addition, Zaka *et al*. (2020) recognized the severe psychological suffering in terms of existential stress and moral injury (Williamson *et al*., 2020) correlated with the loss of many patients, colleagues or loved ones every day.

However, the newly trained healthcare staff for the intensive care unit typically does not have enough psychological training to deal with challenging working environments which that eventually lead them to face immensely challenging circumstances in terms of anger control and existential stress. Under such demeaning circumstances, the most prevalent stress-reduction techniques (Leyens, 2014) may not be feasible in such dire cases, given the possibility of even more emotional involvement of clinical professionals in the regulations to continue.

## Conclusion

Young doctors experienced psychological distress, emotional vulnerability, and disruptive quality of life in the wake of COVID-19. Pandemic anxiety, fear, and apprehension of being exposed to the virus, becoming a probable disease carrier due to shortage of PPE, long working hours, and more organizational responsibility incited concerns for personal and family safety. Contrary to the negative emotional pretext, positive impacts of the pandemic were observed on interpersonal relationships of doctors, receiving more appreciation, and care from family and loved ones.

Moreover, the commitment to service and sense of benevolence, as well as gratification for community service, were among the incentives operating highly for young doctors in devoting themselves to the fight against pandemic.
